# Elevated pro-BNP and low-grade inflammation are associated with low bone mineral density in systemic sclerosis, a case control study

**DOI:** 10.1186/s13075-026-03888-6

**Published:** 2026-09-01

**Authors:** Kerstin Lillpers, Kristofer Andréasson, Seyed Morteza Najibi, Anthony D Woolf, Roger Hesselstrand, Kristina E Åkesson, Meliha C Kapetanovic

**Affiliations:** 1 Department of Clinical Sciences Lund, Lund University, Lund, Sweden; 2 Department of Rheumatology, Skåne University Hospital, Kioskgatan 5, Lund, 22185 Sweden; 3 Department of Clinical Sciences Malmö, Clinical and Molecular Osteoporosis Research Unit, Lund University, Malmö, Sweden; 4 Department of Orthopedics, Skåne University Hospital, Malmö, Sweden

**Keywords:** Systemic sclerosis, Osteoporosis, Osteopenia, Bone mineral density, ProBNP, Inflammation, BMI, Machine learning

## Abstract

**Background:**

Bone mineral density (BMD) is compromised in patients with systemic sclerosis (SSc) compared to the background population, yet the underlying disease related factors are less known. This study evaluates SSc disease-related features and predictability for BMD along with established risk factors for low BMD in SSc patients compared to controls.

**Methods:**

The study includes 211 SSc patients, 182 women and 29 men (mean age 61 yrs, mean disease duration 9 yrs) regularly followed at the Skåne University Hospital, Lund, Sweden and 505 age- and sex-matched controls. Data collection: BMD hip and spine, BMI, SSc-features, biomarkers and potential risk factor questionnaire.

Multinomial logistic regression analysis was used to calculate BMD odds ratio (OR); multiple linear regression for associations between T-score and SSc-related variables and lifestyle factors; and machine learning to fit a model to predict total hip T-score in SSc.

**Results:**

NT-proBNP and elevated CRP were independently associated with low BMD in SSc patients (p<0.001), as were diffuse cutaneous disease subtype, finger ulcers, vital lung capacity and anti-topoisomerase I antibodies (ATA). BMI, menopausal status, low physical activity and higher medication burden were associated with lower hip T-score. Predictive modeling identified BMI and NT-proBNP as the strongest determinants of hip T-score.

**Conclusion:**

This large case–control study finds elevated NT-proBNP, indicators of low-grade inflammation and of disease severity negatively associated with BMD in SSc patients, in addition to several established risk factors. This highlights the importance of BMD assessment to identify at-risk SSc patients beyond traditional risk stratification.

**Supplementary Information:**

The online version contains supplementary material available at https://doi.org/10.1186/s13075-026-03888-6.

## Background

There is growing evidence of an association between systemic sclerosis (SSc) and reduced bone mass; however, detailed understanding of risk factors for osteopenia and osteoporosis in this population, is still limited [1, 2]. Established risk factors for osteoporosis in the general population include female sex, particularly being postmenopausal [3], older age, low physical activity [4, 5], low body mass index (BMI), malnutrition, family history of osteoporosis [6], smoking [7], inflammation [8] and glucocorticoid intake [9].

In our recent study, women with SSc had low bone mineral density (BMD), including those who were premenopausal. In addition to established population-based risk factors for osteoporosis, finger ulcers and diffuse skin involvement were associated with lower BMD whereas prolonged glucocorticoid use was not [1]. Other studies have reported diverging results regarding the association between disease specific features such as vascular dysfunction, calcinosis, and the diffuse cutaneous subtype (dcSSc) and low BMD [10–13]. Furthermore, biomarkers have not been fully addressed. The presence of anti-topoisomerase antibodies (ATA) has shown association with low BMD in some small studies [12, 14, 15] and others have shown association to anti-centromere antibodies (ACA) [10]. Low vitamin D levels in SSc have shown association with low BMD [12, 14, 16] and with fractures [17]. The association between markers of inflammation and BMD in SSc are inconclusive [16, 17].

Osteoporosis is underdiagnosed and undertreated in general resulting in unnecessary fractures [18]. In patients with SSc, fracture-associated morbidity may be even more pronounced emphasizing the importance of identifying patients at high risk of low BMD. However, the contribution and interaction of established risk factors to low bone density and disease specific factors, in SSc is not fully understood.

Because of the diversity in results of previous studies, including our own, we aim to explore in more depth the contributing factors by using our data in more detail, statistically and by considering further variables.

Beyond group-level associations, understanding how individual patient profiles, with their specific combinations of SSc features and established risk factors, may contribute to their bone health may help clinicians tailor screening decisions to the individual rather than relying solely on population-level risk stratification. The aim of the present study is therefore, to determine the contribution of established risk factors for low BMD and potential disease specific risk factors in a cohort of SSc patients by comparing them to matched controls, both women and men. We also aim to explore whether it is possible to predict hip T-score among SSc patients by a data driven approach.

## Methods

### Participants

Patients with SSc, regularly followed at the Rheumatology Department, Skåne University Hospital in Lund, Sweden and controls from the same region participated, as previously described [1]. Briefly, 211 SSc-patients (182 females and 29 males) were enrolled and 505 age and sex matched controls (436 women and 69 men). All SSc-participants fulfilled the 2013 ACR/EULAR classification criteria for SSc [19]. Clinical examination was performed on all patients at inclusion [1]. Data on antibody status, pulmonary function tests with latest vital capacity (VC), calcinosis on first x-ray of hands and disease duration from first non-Raynaud’s symptom was retrieved from medical records retrospectively. Early disease was defined as < 5 years from first non-Raynaud’s symptom. Young age and early menopause were both defined as < 45 years of age.

### Bone mineral density

Dual energy X-ray Absorptiometry (DXA) examinations were performed in all subjects between September 2016 and June 2019 using the same DXA-equipment (Lunar iDXA, GE Healthcare) as previously described [1]. BMD in g/cm^2^ was assessed at the lumbar spine (LS) (vertebra L1-L4) and total hip (TH). Standard quality control was performed. BMD was described according to the WHO thresholds using T-score; osteopenia T-score − 1.0 ‒ -2.5 and osteoporosis T-score ≤ -2.5 [20].

### Laboratory tests

Blood samples were collected at baseline and used for analysis of inflammatory markers (C-reactive protein (CRP) and erythrocyte sedimentation rate (ESR)), haemoglobin, leukocytes, creatinine and calcium. Anemia was defined as haemoglobin < 134 g/L for men and < 117 g/L for women, and for CRP (cut-off > 3 mg/L). In SSc subjects, cartilage oligomeric matrix protein (COMP) U/L [21], N-terminal pro-brain natriuretic peptide (NT-proBNP) ng/L, parathyroid hormone (PTH) pmol/L and 25-OH vitamin D3 nmol/L were also analyzed.

### Questionnaire

A modified comprehensive questionnaire considered general health, medications, reproductive health, physical activity, alcohol and tobacco use, dairy intake and family history of hip fractures [22] and was completed on the same day as the DXA-measurement. Participants listed all ongoing, regular used medications. Antacids, levothyroxine, anti-osteoporotic agents, disease-modifying antirheumatic drugs (DMARDs) including hydroxychloroquine (HCQ) and glucocorticoids were reported separately. Glucocorticoid use was recorded as “taking glucocorticoids ≥ 3 months regularly” and total months of intake. Smoking was recorded as present (including occasional), former or non-smoker. Recommended physical activity was defined according to WHO [23] which entails that all adults should be physically active for a minimum of 2.5 h per week of at least moderate intensity, while low intensive walks were excluded. Intake of dairy products was recorded in dL of milk/yoghurt/sour milk and/or slices of cheese/day.

### Statistics

Descriptive data are presented as means (standard deviation (SD)), median (range) or as frequency (%).

Data was missing at random, the percentage varying between 0.14% and 4%. The Random Forest was employed for the imputation process using 10 trees repeated for 10 times stratified on case and control groups. After checking the robustness of model parameters, a complete dataset has been used for this report.

For hip DXA, the mean value from both hips was used unless only one hip was available (patients *n* = 6 (2.8%); controls = 16 (3.2%)). Missing values for DXA lumbar spine (one patient), total hip (one patient due to bilateral hip replacement and three controls) were dropped from the analysis. T-scores were used as an outcome variable for all participants. Although, Z-scores are recommended for the clinical interpretation of BMD in premenopausal women and younger men, T-scores were used to enable uniform comparisons across the full adult age range.

Multiple linear regression model was used to explore the associations of potential risk factors of low BMD using T-score as a continuous variable at total hip and spine in both patients and controls. The adjusted and unadjusted coefficients have been calculated for each group separately.

Multinomial logistic regression model was applied to calculate the risk (odds ratios, OR) of having low BMD with a T-scores <-1.00 ─ >-2.5 and T-score of ≤-2.5, respectively. Patients and controls were compared regarding three established risk factors; glucocorticoid use > 3 months, smoking and first degree relative with hip fracture [24] and two potential risk factors; low physical activity defined as physical activity less than 2.5 h per week [25, 26] and elevated CRP as a proxy for inflammation [27]. Adjustment for sex, age, BMI and menopause were performed in both models.

A sensitive analysis was performed of the data with and without individuals on anti-osteoporotic agents.

For predictive modeling of total hip T-score, we considered all potential predictors from Table 1, including an interaction term for age and gender, to build a model on the training data (80%) and assess its performance on the test data (20%). We employed elastic-net regularized linear regression [28], decision tree, random forest, and Extreme Gradient Boosting (XGboost) in the procedure. Hyperparameter tuning was performed using 500 random grid search for each model, and model performance was evaluated using 5-fold cross-validation stratified by sex. The final model was selected using the best Root Mean Squared Error (RMSE). During calibration, a systematic miscalibration was observed, which was resolved by post-hoc recalibration with a generalized additive model (GAM) using cubic splines for training-set predictions, then applied to test data. The performance was reported using RMSE, Mean Absolute Error (MAE), and R-squared (R²), evaluated on both training and test sets before and after calibration. The local model-agnostic explainer was computed using Shapley values for the final model, and a global explanation of variable importance, based on RMSE degradation from feature and sample permutation, was reported.

**Table 1:** Baseline data of SSc-patients and age- and sex-match controls and disease features for SSc-patients

	Women		Men	
	**SSc** (*n* = 182)	**Controls** (*n* = 436)	**SSc** (*n* = 29)	**Controls** (*n* = 69)
Age, years mean (SD)	61.3 (12.9)	62.1 (12.6)	62.2 (16.7)	63.5 (15.5)
BMI, kg/m^2^ mean (SD)	24.9 (4.6)	26.1 (5.0)	26.1 (3.7)	27.40 (4.2)
BMI < 20 kg/m^2^%	11.0 (20/182)	5.3 (23/436)	3.4 (1/29)	1.4 (1/69)
Menopause, %	86.7 (157/181)	81.0 (353/436)	NA	NA
Menopausal age, years (SD)	48.7 (5.23)	49.8 (4.89)		
Menopause ≤ 45 years, %	18.8 (34/181)	12.4 (54/436)		
Smoker, %	26.4 (48/182)	22.7 (99/436)	14.3 (4/28)	23.2 (16/69)
Former smoker, %	36.3 (66/182)	37.6 (164/436)	50.0 (14/28)	39.1 (27/69)
Non-smoker, %	37.4 (68/182)	39.7 (173/436)	35.7 (10/28)	37.7 (26/69)
Alcohol, g/year mean (SD)	1782 (2312)	2503 (2848)	3556 (4187)	5435 (6250)
Abstainer, %	21.0 (38/181)	11.8 (51/434)	14.3 (4/28)	7.2 (5/69)
Alcohol ≥ 36 g/day (3 glass) %	0.6 (1/180)	0.9 (4/431)	7.4 (2/27)	8.7 (6/69)
Hip fracture, 1st degree relative %	14.5 (23/159)	18.8 (72/384)	19.0 (4/21)	18.8 (12/64)
No medication use, %	0.6 (1/181)	24.3 (106/436)	3.4 (1/29)	30.4 (21/69)
Medications, total mean (SD)	7.9 (4.7)	2.4 (2.8)	6.4 (4.7)	2.7 (2.8)
DMARDs incl hydrochloroquine, %	30.4 (55/181)	1.4 (6/436)	27.6 (8/29)	1.4 (1/69)
Antacids, %	90.1 (163/181)	9.4 (41/436)	65.5 (19/29)	14.5 (10/69)
Levothyroxine, %	15.5 (28/181)	9.6 (42/436)	3.4 (1/29)	1.4 (1/69)
Anti-osteoporotic agents, %	6.1 (11/181)	2.5 (11/436)	3.4 (1/29)	1.4 (1/69)
Corticosteroids, ever, %	44.0 (80/182)	8.0 (35/436)	55.2 (16/29)	5.8 (4/69)
Corticosteroids, ≥ 3 months^1^, %	23.1 (42/182)	2.8 (12/436)	41.4 (12/29)	2.3 (2/69)
Dairy free diet, %	2.7 (5/182)	3.7 (16/436)	10.3 (3/29)	4.3 (3/69)
Dairy product^2^, mean (SD)	6.14 (3.63)	5.82 (3.67)	7.38 (5.56)	5.64 (3.23)
Low physical activity < 2.5 h/week^3^, %	55.4 (98/177)	43.5 (186/428)	46.4 (13/28)	60.3 (41/68)
P-CRP, mg/L, mean (SD), median (range)	4.2 (8.0), 2.0 (73.4)	2.2 (3.2), 1.1 (31.4)	2.8 (2.8), 1.5 (9.4)	2.2 (3.0), 1.3 (21.4)
P-CRP > 3, mg/L, %	34.1 (62/182)	18.6 (77/436)	31.0 (9/29)	22.4 (15/69)
B-ESR, mm, mean (SD)	25.1 (17.5)	18.6 (12.75)	18.1 (11.5)	12.1 (9.4)
B-Haemoglobin, g/L, mean (SD)	129.3 (11.3)	136.3 (10.1)	137.5 (12.2)	150.9 (10.8)
B-Haemoglobin, anemia^4^%	12.1 (22/182)	2.9 (12/436)	34.5 (10/29)	7.5 (5/69)
B-Leukocytes, 10^9^/L, mean (SD)	7.1 (2.3)	6.5 (1.8)	7.0 (1.9)	6.5 (1.4)
P-Creatinine, mmol/L, mean (SD)	72.0 (30.1)	69.4 (14.8)	88.5 (37.8)	86.3 (18.1)
P-Calcium, mmol/L, mean (SD)	2.4 (0.1)	2.4 (0.9)	2.3 (0.1)	2.4 (0.1)
Years from diagnosis, mean (SD)	9.5 (7.9)	NA	7.4 (8.7)	NA
Years from first non-Raynaud’s symptoms, mean(SD)	13.2 (9.0)		9.1 (8.8)	
lcSSc^5^, *n* (%)	150 (82.4)		20 (69.0)	
dcSSc^6^, *n* (%)	32 (17.6)		9 (31.0)	
Finger ulcers, *n* (%)	14 (7.7)		4 (13.8)	
Telangiectasias, *n* (%)	110 (60.4)		16 (57.1)	
Pitting scars, *n* (%)	71 (39.0)		9 (32.1)	
mRSS^7^, mean (SD), median (range)	3.72 (5.92), 2.0 (45.0)		6.40 (8.80), 4.0 (37.5)	
Vital lung capacity, % of expected, mean (SD)	94.8 (17.6)		88.9 (14.7)	
Calcinosis on first X-ray of the hands, *n* (%)	49 (28.5)		6 (22.2)	
Antibodies ACA^8^, n (%)	77 (42.3)		5 (17.2)	
ARA^9^, *n* (%)	15 (8.2)		4 (13.8)	
ATA^10^, *n* (%	22 (12.1)		4 (13.8)	
ANA^11^, *n* (%)	56 (30.8)		11 (37.9)	
ANA negative, *n* (%)	12 (6.6)		5 (17.2)	
S-25-OH vitamin D, nmol/L mean (SD)	68.7 (23.1)		60.9 (19.9)	
P-Albumin, g/L mean (SD)	39.5 (3.3)		40.0 (3.6)	
P-PTH, pmol/L mean (SD), median (range)	5.5 (7.0), 4.4 (88.6)		5.1 (2.6), 4.0 (37.5)	
S-COMP, U/L mean (SD)	11.2 (4.5)		12.8 (4.1)	
P-NT-proBNP, ng/L mean (SD), median (range)	432.2 (1328.0), 143.5 (15442)		300.3 (526.5), 115.0 (2690)	

^1^ Regular use, ^2^ milk/yoghurt/sour milk (dL/day) and/or slices of cheese/day, ^3^ Excluding walks, ^4^ B-Hemoglobin < 117 g/L women and < 134 g/L men, ^5^ Limited cutaneous systemic sclerosis, ^6^ diffuse cutaneous systemic sclerosis,^7^ Modified Rodnan Skin Score, ^8^ Anti-Centromer antibody; ^9^ anti-RNA polymerase III antibody; ^10^ Anti-Topoisomerase antibody; ^11^ ANA except ACR, ARA, ATA,^12^ Cartilage Oligomeric Matrix Protein

Nominal significance was determined as *p* ≤ 0.05. The statistical analysis was performed using R Core Team 4.4.2 (2024).

## Results

### Characteristics of the participants

The baseline characteristics of patients and controls are summarized in Table 1.

The mean age of SSc patients were 61.3 years (range 21.2–86.0) for women and 62.2 years (range 29.4–85.7) for men. Women with SSc had lower BMI and a higher proportion had low physical activity compared to their controls. Anemia was present in 12% of women and 35% of men with SSc, but less common in controls. CRP was elevated in approximately one third of SSc patients (women 34%, men 31%) compared to one fifth of controls (Table 1).

Daily medications were used by almost all SSc patients, in contrast to controls (daily medication, 76% women; 70% men). Corticosteroid use daily for > 3 months was more frequent among patients (SSc 23% and controls 3%, respectively).

Osteoporosis was self-reported by 7.7% (*n* = 14) of women with SSc and 5.0% (*n* = 22) of controls (Supplementary, Table S1), with anti-osteoporotic treatment being reported by 6.1% (*n* = 12) patients and 2.5% (*n* = 12) controls (data not shown). All were taking bisphosphonates except two SSc-patients who were on denosumab and one on strontium ranelate. Of all treated, only 2 patients and 3 controls had total hip and/or lumbar spine T-score ≤ -2.5 and 7 patients and 5 controls had T-score − 1.0 ‒ -2.5. The rest had normal BMD.

### Disease characteristics

SSc disease characteristics are presented in Table 1. Disease duration from first non-Raynaud symptom were 13.2 (9.0) and 9.1 (8.8) years, women and men, respectively. Most patients had limited cutaneous SSc (lcSSc) (82.4% women; 69.0% men). ACA was most common among women (42%) and anti-nuclear antibodies (ANA, without ACA, ATA and anti-RNA polymerase III antibodies (ARA)) most common among men (39%). Pulmonary function (VC) was within reference range in the majority of patients, although slightly lower in men. DMARDs including hydroxychloroquine were ongoing in approximately 30%.

### Association of potential risk factors to T-score in total hip and spine for patients and controls

A lower hip T-score was associated with each unit increase in CRP (B -0.04; *p* < 0.001) and ESR (B -0.01; *p* = 0.001) in SSc patients, the opposite was seen for hemoglobin levels (Table 2). Low spine T-score was significantly associated with lower values of hemoglobin but not with CRP (Table 2). No such associations were observed among controls.

**Table 2:** Multiple linear regression analysis for associations between T-score and potential risk factors for low BMD

	Adjusted hip						Adjusted spine					
	SSc			Control			SSc			Control		
	B	95% CI	*P*	B	95% CI	*p*	B	95% CI	*p*	B	95% CI	*P*
No medication	0.75	-0.64, 2.1	0.3	-0.02	-0.20, 0.15	0.8	0.00	-1.8, 1.8	> 0.9	-0.11	-0.40, 0.18	0.5
Total medications	**-0.04**	**-0.07**,** -0.02**	**0.002**	0.00	-0.03, 0.03	> 0.9	-0.03	-0.06, 0.01	0.2	**0.05**	**0.00**,** 0.10**	**0.05**
DMARDs incl HCQ	-0.23	-0.51, 0.06	0.12	-0.22	-0.90, 0.45	0.5	-0.04	-0.41, 0.32	0.8	-0.55	-1.6, 0.53	0.3
Corticosteroids^1^	-0.05	-0.35, 0.26	0.8	-0.20	-0.68, 0.28	0.4	0.01	-0.38, 0.40	> 0.9	0.23	-0.54, 0.99	0.6
Antacida	-0.21	-0.51, 0.10	0.2	0.02	-0.25, 0.29	0.9	-0.13	-0.52, 0.25	0.5	0.40	-0.04, 0.84	0.072
Hip fracture, relative^2^	-0.23	-0.63, 0.16	0.2	-0.14	-0.35, 0.07	0.2	-0.09	-0.60, 0.42	0.7	0.03	-0.31, 0.37	0.9
Smoking^3^	-0.20	-0.51, 0.10	0.2	**-0.19**	**-0.39**,** 0.00**	**0.05**	**-0.45**	**-0.83**,** -0.06**	**0.022**	-0.02	-0.33, 0.29	> 0.9
Dairy product/day^4^	0.01	-0.02, 0.04	0.6	**0.02**	**0.00**,** 0.05**	**0.038**	0.01	-0.03, 0.05	0.7	0.01	-0.02, 0.05	0.5
Low physical activity^5^	**-0.29**	**-0.55**,** -0.03**	**0.031**	-0.03	-0.19, 0.13	0.7	-0.25	-0.58, 0.09	0.14	-0.09	-0.34, 0.17	0.5
P-CRP (mg/L)	**-0.04**	**-0.06**,** -0.02**	**< 0.001**	0.01	-0.02, 0.03	0.6	-0.02	-0.05, 0.01	0.11	0.04	-0.00, 0.08	0.066
B-ESR (mm)	**-0.01**	**-0.02**,** 0.00**	**0.001**	0.00	-0.01, 0.01	0.8	-0.01	-0.02, 0.00	0.2	0.01	-0.01, 0.02	0.3
B-Haemoglobin (g/L)	**0.02**	**0.01**,** 0.03**	**0.001**	0.00	-0.01, 0.01	0.8	**0.02**	**0.00**,** 0.03**	**0.009**	-0.01	-0.02, 0.00	0.2
B-Leukocytes (10*9/L)	**-0.06**	**-0.12**,** 0.00**	**0.04**	-0.03	-0.08, 0.01	0.15	-0.05	-0.13, 0.02	0.2	**0.08**	**0.00**,** 0.15**	**0.047**
P-Creatinine (mmol/L)	0.00	-0.01, 0.00	0.11	0.00	0.00, 0.01	0.2	0.00	-0.01, 0.01	0.9	0.00	0.00, 0.01	0.3
P-Calcium (mmol/L)	-0.67	-2.0, 0.64	0.3	-0.29	-1.2, 0.61	0.5	0.37	-1.30, 2.00	0.7	-0.53	-2.0, 0.91	0.5

Regression analysis adjusted for sex, age, BMI and menopause. Unadjusted analysis can be found in Supplement Table S2. ^1^ Regular for > 3 months, ^2^ 1st degree relative, ^3^ Including occasional, ^4^ milk/yoghurt/sour milk (dL/day) and/or slices of cheese/day, ^5^ < 2.5 h/week excluding walks. Bold text: p ≤ 0.05

Lower hip T-score was also associated with higher use of medications in patients (Table 2).

Smoking was not associated with lower hip T-scores but was with a lower spine T-score in SSc patients (Table 2).

For patients, insufficient physical activity showed a negative impact on T-scores at the hip, (hip: B = -0.29, *p* = 0.031), with a similar trend for the spine although the associations did not reach statistical significance (B = -0.25, *p* = 0.14) (Table 2).

### Comparing established and potential risk factors for low BMD in subgroups of patients and controls

In a clinical context, all participants were categorized in subgroups according to their lowest BMD value in hip and/or spine (Supplement Table S2). Women (patients and controls) with T-score ≤ -2.5 were older, had lower BMI and were more often postmenopausal, compared to individuals with normal BMD. CRP values were higher in SSc patients, with the highest values in SSc women with the lowest BMD.

The results of the adjusted and unadjusted regression analysis regarding three established and two potential predictors of low BMD for each group separately are presented in (Supplementary Table S4). In patients, CRP (log) increased the OR of having the lowest BMD (T-score ≤-2.5) (OR 1.87; 95% CI 1.15–3.03) as did smoking (OR 3.12; 95% CI 1.07–9.13).

### Multiple linear regression model on T-score in total hip and spine for SSc-patients including disease features

Lower hip T-score was independently associated with higher levels of NT-proBNP (log) (B = − 0.31, *p* < 0.001), CRP (log) (B = − 0.29, *p* < 0.001), PTH (log) (β = − 0.57, *p* = 0.001), dcSSc, ATA and finger ulcers in addition to greater medication intake after adjustment (Table 3). Results of the unadjusted analysis are displayed in Supplementary Table S5.

**Table 3:** Associations between T-score and potential risk factors for low BMD in SSc-patients

	Total hip			Lumbar spine		
	B	95% CI	*p*	Β	95% CI	*p*
Disease duration (years)	0.00	-0.02, 0.02	> 0.9	0.01	-0.01, 0.03	0.3
Diffuse cSSc	**-0.38**	**-0.72**,** -0.04**	**0.03**	0.04	-0.40, 0.47	0.9
Finger ulcers	**-0.59**	**-1.1**,** -0.12**	**0.014**	-0.22	-0.82, 0.39	0.5
Telangiectasia	-0.21	-0.48, 0.06	0.12	-0.04	-0.39, 0.31	0.8
Pitting scars	-0.05	-0.33, 0.24	0.8	-0.02	-0.38, 0.34	> 0.9
mRSS	-0.02	-0.04, 0.00	0.1	-0.02	-0.04, 0.01	0.3
Latest vital lung capacity (%)	**0.01**	**0.00**,** 0.02**	**0.012**	0.00	-0.01, 0.01	0.5
Calcinosis on first hand x-ray	-0.12	-0.42, 0.18	0.4	0.17	-0.21, 0.55	0.4
Antibodies						
ACA (reference)	—	—		—	—	
ARA	-0.37	-0.85, 0.11	0.13	-0.27	-0.89, 0.35	0.4
ATA	**-0.54**	**-0.97**,** -0.11**	**0.015**	0.12	-0.43, 0.68	0.7
ANA pos (not ACA, ARA, ATA)	-0.04	-0.36, 0.28	0.8	0.36	-0.05, 0.78	0.088
ANA neg	0.24	-0.29, 0.76	0.4	0.16	-0.50, 0.81	0.6
Corticosteroid duration (months)	**0.00**	**0.00**,** 0.01**	**< 0.001**	0.00	0.00, 0.01	0.12
P-CRP (log) (mg/L)	**-0.27**	**-0.40**,** -0.14**	**< 0.001**	**-0.19**	**-0.36**,** -0.02**	**0.025**
S-25-OH Vitamin D (nmol/L)	**0.01**	**0.00**,** 0.01**	**0.049**	**0.01**	**0.00**,** 0.02**	**0.04**
P-Albumin (g/L)	0.04	0.00, 0.08	0.073	0.02	-0.03, 0.07	0.5
P-PTH (log) (pmol/L)	**-0.58**	**-0.90**,** -0.26**	**< 0.001**	**-0.49**	**-0.90**,** -0.02**	**0.022**
S-COMP (U/L)	0.00	-0.03, 0.03	0.8	0.02	-0.02, 0.06	0.3
P-NT-proBNP (log) (ng/L)	**-0.31**	**-0.44**,** -0.18**	**< 0.001**	-0.12	-0.30, 0.05	0.2

Regression analysis adjusted for sex, age, BMI and menopause. Unadjusted analysis can be found in Supplement Table S5. Bold text: p ≤ 0.05

Higher lung VC and S-25-OH Vitamin D were positively associated with hip T-score.

Lower spine T-scores were associated with elevated CRP levels and PTH but with none of the disease characteristics (Table 3). Higher spine T-score was associated with higher vitamin D levels.

### Sensitive analysis

Sensitivity analysis was performed excluding individuals on anti-osteoporotic treatment (*n* = 24). The results changed only minimally in the regression analyses. Minor changes were observed in the OR analysis where the adjusted OR for having a T-score < − 1.0 to > − 2.5 for low physical activity changed from 2.09 (*p* = 0.029) to 1.87 (*p* = 0.072), and the OR for having a T-score ≤ − 2.5 for CRP changed from 1.87 (*p* = 0.012) to 1.68 (*p* = 0.045).

### Predictive modeling on T-score in total hip among SSc-patients

**Fig. 1:**
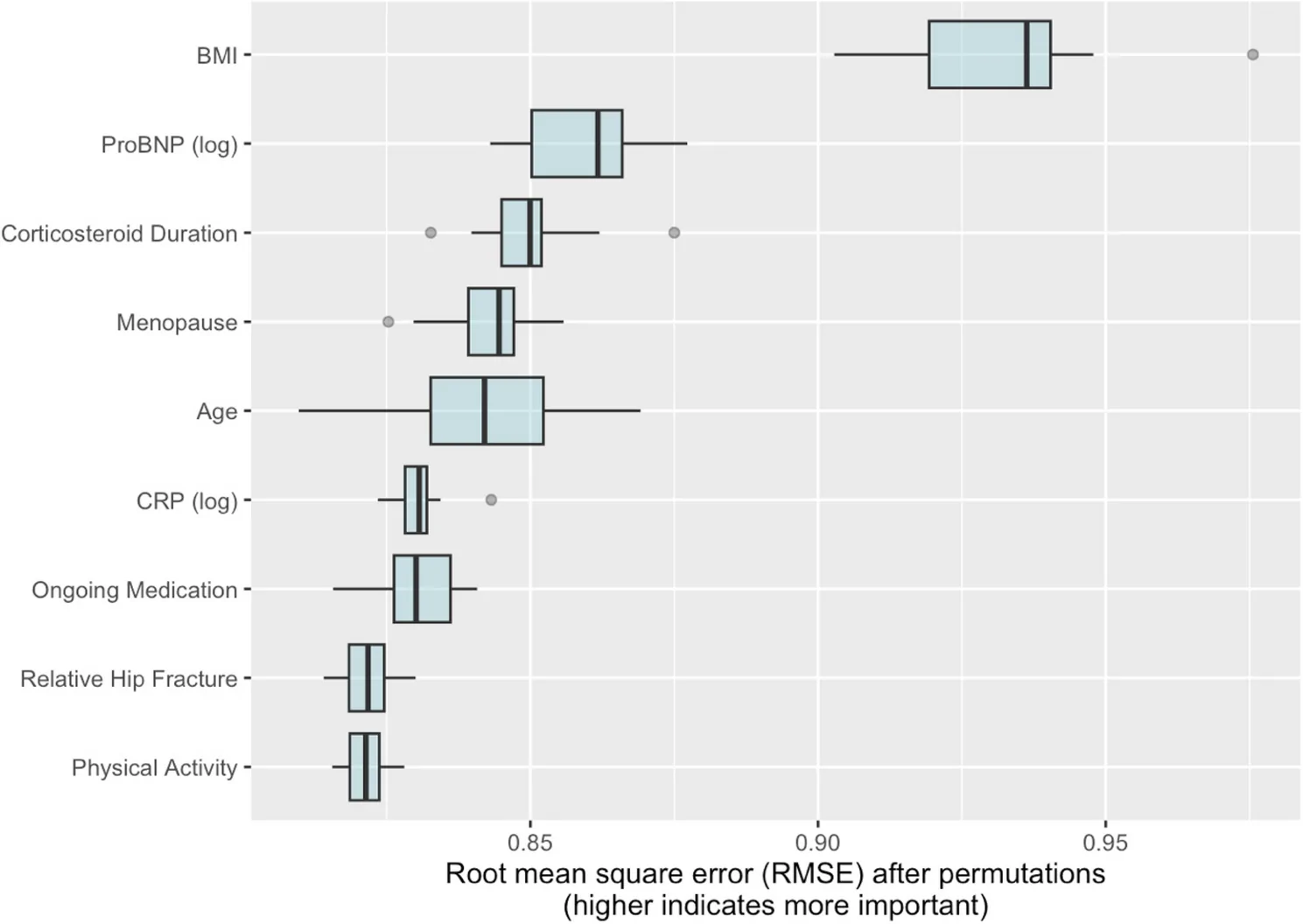
The global model’s variable importance for the nine most important factors. Age: Years, Corticosteroid duration: months, Ongoing Medication: number of, Relative hip fracture: Yes, Physical activity: Insufficient exercise < 2.5 h/week excluding walks

**Fig. 2:**
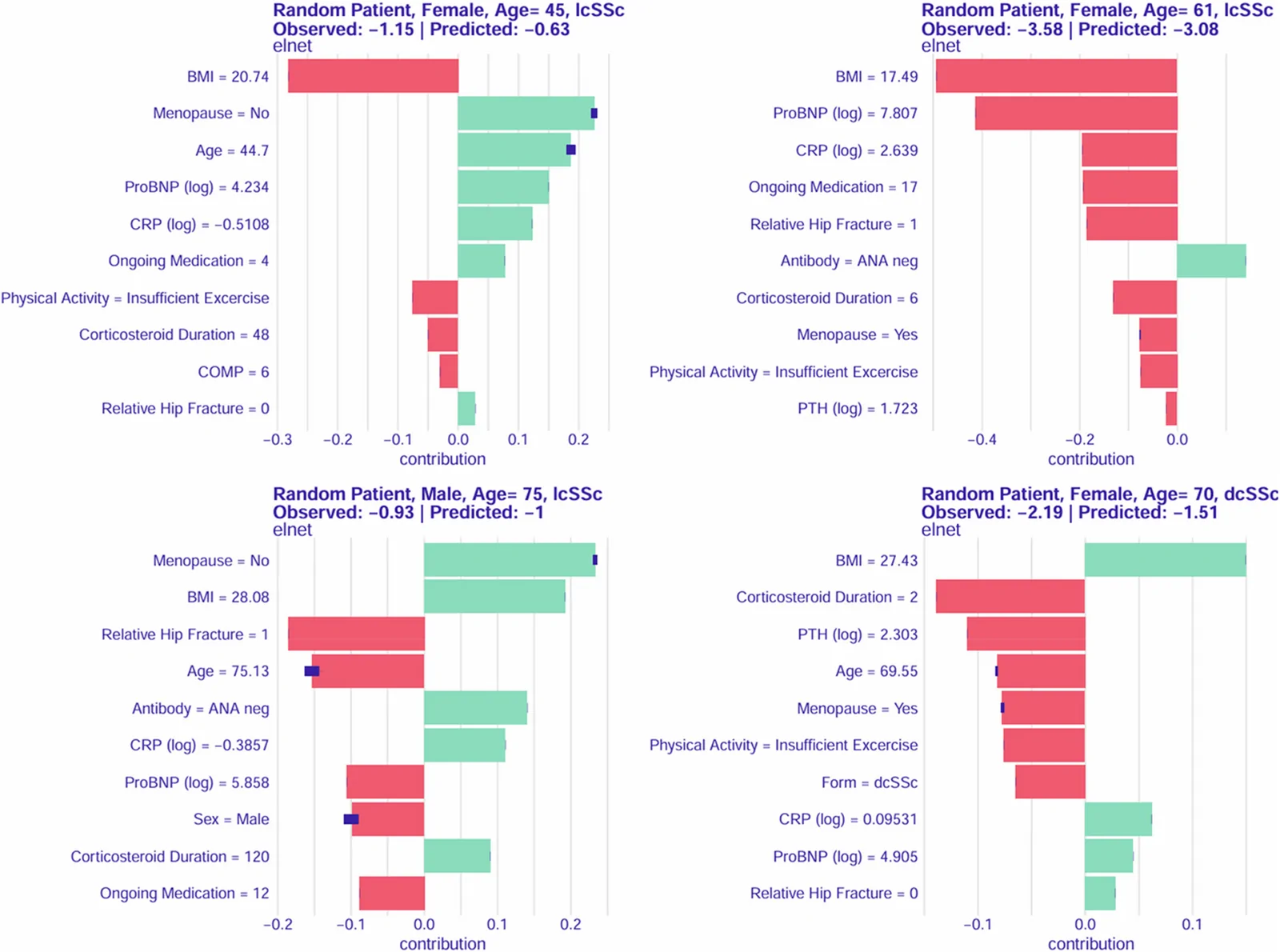
The local Shapley explanations for four random individual patients illustrate the feature contributions to their predicted total hip T-scores. The observed total hip T-score is also displayed. For each selected patient, the Shapely plots visualize the additive contributions of variables relative to a baseline (intercept) prediction. Positive contributions increase the predicted value, while negative contributions decrease it. Age: Years, Corticosteroid duration: months, Ongoing Medication: number of, Relative hip fracture: 0 = No, 1 = Yes, Physical activity: Insufficient exercise < 2.5 h/week excluding walks

The regularized regression using elastic net achieved the lowest RMSE (0.97) on testing data and has been selected as the final model with the tunned parameters. The model’s performance in training and test, before and after calibration reported in Table 4. The recalibrated model improved the alignment between the predicted and observed score depicted in Supplementary Figure S1. The final model confirmed the following nine variables, in order of importance, for predicting total hip T-scores: BMI, NT-proBNP, duration of corticosteroid use, menopausal status, age, CRP, total medication intake, first degree relative with hip fracture and physical activity (Fig. 1). The strongest predictability was observed with BMI and NT-proBNP. Coefficients for most of the other variables shrunk to zero, indicating limited additional predictive value under penalization. The local Shapley explanations for four random patients illustrate the feature contributions to their predicted total hip T-scores (Fig. 2) using the selected model. The figure illustrates how the same model generates individual predictions based on distinct covariate profiles. For example, male age 75, with high BMI and low CRP, receives a prediction close to the population mean, whereas female age 61, characterized by low BMI, elevated NT-proBNP and CRP shows additive negative contributions from multiple features, yielding a substantially lower predicted T-score.

**Table 4:** The final model´s performance metrics, before and after calibration

Data set	Calibration	RMSE	MAE	*R* ^2^
Train	No	0.81	0.64	0.44
Test	No	0.97	0.77	0.45
Train	Yes	0.80	0.63	0.44
Test	Yes	0.95	0.75	0.45

*RMSE* Root Mean Squared Error, *MAE* Mean Absolute Error, *R*² R-squared

## Discussion

In this cohort of patients with systemic sclerosis, even modest elevations in systemic inflammatory markers and indicators of more severe disease were significantly associated with reduced BMD, in addition to established risk factors.

Higher values of NT-proBNP were independently associated with lower T-scores among SSc-patients. To the best of our knowledge, this association has not previously been reported in patients with SSc. Higher values of NT-proBNP have been reported in association with low BMD in patients with heart failure [29]. Cardiac manifestations are overrepresented [30–32] in SSc. They can be heterogeneous when present [29] and contribute to impaired physical performance in affected patients.

Markers reflecting inflammation, elevated levels of CRP and ESR and lower levels of hemoglobin were associated with low BMD in this study. While not markedly altered, it is in line with low grade inflammation, which is a known risk factor for osteoporosis [8, 30]. In a large cohort of 75-year-old women, persistently elevated CRP was associated with bone loss [27]. Although a single CRP value may be an inadequate indicator of an individual’s sustained inflammatory status, it may still offer useful contextual information especially when this is accompanied by other markers of inflammation such as ESR and low hemoglobin levels.

SSc is generally characterized as a low-grade inflammatory condition, in contrast to diseases such as rheumatoid arthritis, where systemic inflammation is more pronounced and is a recognized risk factor of osteoporosis [31]. Nevertheless, this study suggests that low-grade inflammation may also contribute to bone health in SSc. The association between higher CRP levels and more severe morbidity and increased mortality in SSc has previously been reported [32]. The association of a small elevation in CRP as observed in this study on bone density highlights both the need for DXA screening in SSc patients with elevated indicators of inflammation and the importance of treating the residual inflammation in these patients.

The association between the higher number of concomitant medications and lower hip T-score is also an important finding. Although there was no significant difference in comorbidities between SSc patients and their controls, the higher number of concomitant medications probably indicate the more severe morbidity burden among SSc patients.

In addition, disease features known to be associated with a more severe SSc-phenotype, such as dcSSc and ATA, finger ulcers and impaired lung capacity, as well as elevated pro-BNP and low-grade inflammation suggest that greater disease burden is associated with lower T-score in SSc. Unfortunately, a composite SSc disease activity measure was not validated at the study initiation. Some of the required variables (e.g. in the EUSTAR Activity Index [33]) were not available retrospectively. Therefore, the potential impact of overall SSc disease activity on bone health may not have been fully captured.

In healthy individuals, higher levels of physical activity are associated with increased total hip BMD in both men and women [4]. In our analysis, the results indicate a trend toward lower BMD among less active [23] individuals with SSc. Further research including more detailed data on how physical activity influence BMD in patients with SSc is needed, especially since physical activity is a potentially modifiable factor.

In recent years, studies using prediction models by machine learning to estimate T-score [34] and osteoporosis [35, 36] have begun to emerge. To explore the possibility to predict total hip T-score based on our data we constructed a predictive model based on both established risk factors for low BMD and potential variables available for SSc-patients. The local Shapley explanations (Fig. 2) are intended to open a discussion about the clinical utility of individualized prediction in SSc. Unlike global variable importance, which reflects average contributions across all patients, local explanations reveal that the same predictor, such as corticosteroid duration, may contribute differently to an individual’s predicted T-score depending on their broader clinical picture. The heterogeneity in feature contributions underscores that low bone density in SSc is not driven by a few dominant factors, but by the interplay of disease-specific and traditional risk factors that vary between individuals. The model demonstrated reasonable performance in predicting hip T-scores overall, although some overfitting was observed at both the lower and upper extremes. Importantly, from a clinical standpoint, the principal objective is to identify individuals at risk of low BMD rather than estimate the exact T-score. In this regard, the model performed with greater accuracy. However, as illustrated in Fig. 2, the model struggled to predict T-scores in specific cases. The limited sample size for these types of analyses likely contributed to reduced predictive precision and highlighted the need for larger datasets, potentially achievable through multicenter collaboration. The models also need validation in independent cohorts. Addition of longitudinal data are needed to develop and validate models for predicting bone loss over time.

For several of the selected variables, the overlap between the regression analysis and the final prediction model shows high level of agreement between the potential predictors of total hip T-score in SSc. In the prediction model, NT-proBNP, CRP, duration of corticosteroid use, and total medication intake, together with established factors, contributed to deterioration in bone health in SSc.

A strength of the present single-center case–control study is the inclusion of over 200 prevalent SSc cases and more than twice as many age-matched controls from the general population, making it one of the largest studies to date on risk factors for low BMD in SSc. The cohort includes men—among whom SSc is rare—and younger women, with approximately 70% of all SSc patients attending our department represented.

Some limitations should be noted. Retrospective data collection via questionnaires introduces potential recall bias, although this is likely comparable between patients and controls. The exact data on corticosteroid dosage and cumulative exposure were not collected in the present study. However, corticosteroids prescribed in SSc are typically at low doses due to the risk of renal crisis [37]. Higher single doses of pulse therapy is very rarely used in SSc. Of all patients, only two received Rituximab every sixth months where a higher dose of corticosteroids was used as premedication. In our study there was no association between glucocorticoid use and low BMD when DXA was performed on all patients, in contrast to a retrospective study from France [15] recently reporting such an association in SSc. This discrepancy may partly reflect an indication bias in retrospective cohorts, where patients on glucocorticoids are more likely to be referred to DXA, inflating the apparent association. The lack of corticosteroid doses in our study, albeit difficult to accurately estimate because of dosing variability over time, may have influenced the prediction model (Figs. 1 and 2) where the duration of corticosteroid use seems to have a non-linear contribution to the prediction.

Biomarkers, such as NT-proBNP, CRP and PTH, were only measured once which limits their interpretability. Furthermore, cumulative inflammation or cardiac impairment over time were not captured even though inflammation parameters were higher for patients than for controls. Subsequently, detailed comorbidity data were not gathered—only predefined diagnoses were included in the questionnaire (Supplementary, Table S1). Associations of NT-proBNP and CRP are only indicative and should be interpreted with caution due to lack of detailed comorbidity data and longitudinal alterations. The observed associations involving PTH should be considered exploratory. Although, calcium and 25-hydroxyvitamin D were available, the study was not designed to evaluate calcium–PTH homeostasis or the appropriateness of the PTH response.

## Conclusion

This large case–control study finds that elevated NT-proBNP, low-grade inflammation and indicators of disease severity are negatively associated with BMD. In addition, it confirms that several established risk factors associated with low BMD, including low BMI and being post-menopausal influence bone health in patients with SSc. Data driven models in SSc for BMD prediction could potentially support clinical reasoning: flagging patients whose risk is driven primarily by modifiable factors versus those where non-modifiable disease severity dominates.

These findings emphasize the importance of addressing bone health, not only in patients with established risk factors but in those with more pronounced SSc-disease for BMD assessment, regardless of age, sex and menopausal status and encourage further investigations regarding prediction modeling in SSc.

## Supplementary Information


Supplementary Material 1.


## Data Availability

The data underlying this article will be shared on reasonable request to the corresponding author.

## Abbreviations

SSc: Systemic sclerosis
DMD: Bone mineral density
dcSSc: Diffuse cutaneous systemic sclerosis
ATA: Anti-topoisomerase antibodies
ACA: Anti-centromere antibodies
ACR: American College of Rheumatology
EULAR: European Alliance of Associations for Rheumatology
VC: Vital capacity
DXA: Dual energy x-ray absorptiometry
LS: Lumbar spine
TH: Total hip
WHO: World Health Organization
CRP: C-reactive protein
ESR: Erythrocyte sedimentation rate
COMP: Cartilage oligomeric matrix protein
NTpro-BNP: N-terminal pro-brain natriuretic peptide
PTH: Parathyroid hormone
DMARSs: Disease modifying anti-rheumatic drugs
HCQ: Hydroxychloroquine
OR: Odds ratio
XGboost: Extreme gradient boosting
RMSE: Root mean squared error
GAM: Generalized Additive Model
MAE: Mean absolute error
R^2^: R-squared
lcSSc: Limited cutaneous SSc
ANA: Anti-nuclear antibodies
ACR: Anti-RNA polymerase III antibodies
