# Supplementary material for: Elevated pro-BNP and low-grade inflammation are associated with low bone mineral density in systemic sclerosis, a case control study
Source: Arthritis Res Ther. 2026 Sep 1;28:160. doi: 10.1186/s13075-026-03888-6 (PMC13531976; doi:10.1186/s13075-026-03888-6)
Supplement: Supplementary file 1 [file 13075_2026_3888_MOESM1_ESM.docx]

**Supplementary**

**Table S1.** Comorbidities among patients with SSc and matched controls, collected from questionnaires

| Comorbidities % (n) | Women | | Men | |
| --- | --- | --- | --- | --- |
|  | **SSc** n=182 | **Controls** n=436 | **SSc** n=29 | **Controls** n=69 |
| Diabetes | 7.1 (13) | 8.0 (35) | 6.9 (2) | 13.0 (9) |
| Thyroide disease | 12.6 (23) | 11.0 (48) | - | - |
| Lactose intolerance | 3.8 (7) | 3.9 (17) | 3.4 (1) | 1.4 (1) |
| Gluten intolerance | 1.6 (3) | 3.0 (13) | 6.9 (2) | - |
| Inflammatory bowel disease | 0.5 (1) | 1.6 (7) | 3.4 (1) | 3.0 (2) |
| Asthma | 8.8 (16) | 9.8 (43) | 3.4 (1) | 5.8 (4) |
| Allergy | 20.9 (38) | 19.7 (86) | 17.2 (5) | 18.8 (13) |
| Rheumatoid arthritis | NA | 2.1 (9) | NA | - |
| Multiple sclerosis | 1.1 (2) | - | - | 1.4 (1) |
| Epilepsy | 1.1 (2) | 0.2 (1) | - | - |
| Migraine | 15.4 (28) | 19.7 (86) | 3.4 (1) | 5.8 (4) |
| Tumor | 13.2 (24) | 11.0 (48) | 10.3 (3) | 10.1 (7) |
| Hypertension | 22.5 (41) | 31.0 (135) | 37.9 (11) | 30.0 (20) |
| Osteoporosis | 7.7 (14) | 5.0 (22) | - | - |
| Polycystic ovary syndrome | 2.2 (4) | 1.6 (7) | NA | NA |
| Anorexia/bulimia | 3.3 (6) | 3.7 (16) | - | - |

**Table S2.** Unadjusted linier regression analysis for associations between T-score and potential risk factors for low BMD.

|  | **Unadjusted hip** | | | | | | **Unadjusted spine** | | | | | |
| --- | --- | --- | --- | --- | --- | --- | --- | --- | --- | --- | --- | --- |
| **Characteristics** | **SSc** | | | **Control** | | | **SSc** | | | **Control** | | |
|  | **B** | **95% CI***^1^* | **P** | **B** | **95% CI***^1^* | **p** | **B** | **95% CI***^1^* | **p** | **Β** | **95% CI***^1^* | **P** |
| No medication | 0.90 | -0.66, 2.5 | 0.3 | 0.13 | -0.07, 0.34 | 0.2 | 0.16 | -1.8, 2.1 | 0.9 | -0.02 | -0.32, 0.28 | 0.9 |
| Total medications | -0.05 | -0.08, -0.02 | 0.001 | -0.03 | -0.06, 0.01 | 0.13 | -0.04 | -0.08, 0.00 | 0.083 | 0.04 | -0.01, 0.09 | 0.14 |
| DMARDs incl HCQ | -0.09 | -0.42, 0.24 | 0.6 | -0.38 | -1.2, 0.45 | 0.4 | 0.10 | -0.32, 0.51 | 0.6 | -0.73 | -1.90, 0.47 | 0.2 |
| Corticosteroids^1^ | -0.01 | -0.36, 0.33 | >0.9 | -0.29 | -0.88, 0.30 | 0.3 | 0.11 | -0.32, 0.55 | 0.6 | 0.23 | -0.63, 1.10 | 0.6 |
| Antacida | -0.22 | -0.57, 0.13 | 0.2 | 0.10 | -0.22, 0.43 | 0.5 | -0.17 | -0.61, 0.26 | 0.4 | 0.59 | 0.12, 1.10 | 0.015 |
| Hip fracture, relative^2^ | -0.46 | -0.91, -0.01 | 0.045 | -0.31 | -0.57, -0.05 | 0.019 | -0.26 | -0.83, 0.31 | 0.4 | -0.12 | -0.50, 0.26 | 0.5 |
| Smoking^3^ | -0.30 | -0.65, 0.04 | 0.092 | -0.01 | -0.25, 0.22 | >0.9 | -0.55 | -0.99, -0.12 | 0.013 | 0.10 | -0.24, 0.43 | 0.6 |
| Dairy products^4^ | -0.01 | -0.05, 0.03 | 0.5 | 0.01 | -0.02, 0.03 | 0.6 | 0.00 | -0.05, 0.05 | >0.9 | 0.00 | -0.04, 0.04 | >0.9 |
| Low physical activity^5^ | -0.28 | -0.59, 0.02 | 0.068 | -0.04 | -0.24, 0.16 | 0.7 | -0.28 | -0.66, 0.11 | 0.2 | -0.06 | -0.34, 0.23 | 0.7 |
| P-CRP (mg/L) | -0.05 | -0.08, -0.03 | <0.001 | 0.02 | -0.01, 0.05 | 0.3 | -0.04 | -0.07, -0.01 | 0.023 | 0.06 | 0.01, 0.10 | 0.011 |
| B-SR (mm) | -0.02 | -0.03, -0.01 | <0.001 | 0.00 | -0.01, 0.01 | 0.4 | -0.01 | -0.02, 0.00 | 0.042 | 0.00 | -0.01, 0.01 | 0.6 |
| B-Haemoglobin (g/L) | 0.03 | 0.02, 0.04 | <0.001 | 0.00 | -0.01, 0.01 | 0.7 | 0.03 | 0.02, 0.05 | <0.001 | 0.00 | -0.01, 0.01 | 0.9 |
| B-Leukocytes (10*9/L) | -0.09 | -0.16, -0.02 | 0.008 | -0.03 | -0.08, 0.03 | 0.3 | -0.07 | -0.16, 0.01 | 0.085 | 0.10 | 0.01, 0.18 | 0.022 |
| P-Creatinine (mmol/L) | -0.01 | -0.01, 0.00 | 0.025 | 0.00 | 0.00, 0.01 | 0.7 | 0.00 | -0.01, 0.01 | 0.9 | 0.01 | 0.00, 0.02 | 0.057 |
| P-Calcium (mmol/L) | -1.5 | -3.0, -0.03 | 0.046 | -1.4 | -2.5, -0.23 | 0.018 | -0.64 | -2.50, 1.20 | 0.5 | -1.9 | -3.40, -0.29 | 0.021 |

*^1^* CI = Confidence Interval

^1^Regular for > 3 months, ^2^1^st^ degree relative, ^3^Including occasional, ^4^milk/yoghurt/sour milk (dL/day) and/or slices of cheese/day, ^5^ <2.5 h/week excluding walks

**Table S3.** SSc patients and match controls in subgroups depending on BMD

| WOMEN | **T-score ≥ -1.00** | | **T-score <-1.00 ─ > -2.5** | | **T-score ≤ -2.5** | |
| --- | --- | --- | --- | --- | --- | --- |
|  | SSc  n=58 | Controls n=204 | SSc  n=94 | Controls  n=184 | SSc  n=30 | Controls  n=48 |
| Mean (SD) |  |  |  |  |  |  |
| Age, years | 54.8 (13.2) | 57.1 (13.1) | 64.0 (11.6) | 65.6 (10.8) | 65.8 (11.9) | 69.9 (8.2) |
| BMI kg/m | 26.5 (4.8) | 27.4 (5.6) | 24.8 (4.4) | 25.3 (4.3) | 21.8 (3.7) | 23.5 (3.0) |
| CRP, mg/L | 3.0 (4.3) | 2.5 (3.9) | 3.5 (4.6) | 2.2 (2.7) | 8.9 (16.3) | 1.4 (1.4) |
| n (%) |  |  |  |  |  |  |
| Menopause | 41 (70.7) | 137 (67.2) | 88 (93.6) | 168 (91.3) | 28 (93.3) | 48 (100.0) |
| Corticosteroids^2^ | 12 (21.1) | 7 (3.4) | 21 (22.3) | 2 (1.1) | 9 (31.0) | 3 (6.3) |
| Hip fracture, relative^3^ | 3 (5.2) | 31 (15.2) | 16 (17.0) | 35 (19) | 4 (13.8) | 6 (12.5) |
| Smoking^1^ | 10 (17.2) | 54 (26.3) | 26 (27.7) | 31 (16.8) | 12 (40.0) | 14 (29.2) |
| Low physical activity^4^ | 26 (49.1) | 87 (43.9) | 52 (57.1) | 73 (40.1) | 17 (56.7) | 26 (54.2) |
|  |  | |  | |  | |
| MEN | **T-score ≥ -1.00** | | **T-score <-1.00 ─ > -2.5** | | **T-score ≤ -2.5** | |
|  | SSc  n=14 | Controls  n=44 | SSc  n=14 | Controls  n=24 | SSc  n=1 | Controls  n=1 |
| Mean (SD) |  |  |  |  |  |  |
| Age, years | 60.1 (15.4) | 60.4 (16.5) | 62.8 (18.1) | 69.4 (12.7) | 81.5 | 61.2 |
| BMI, kg/m | 26.0 (2.2) | 28.0 (4.6) | 25.7 (4.6) | 26.3 (3.3) | 32.1 | 28.0 |
| CRP, mg/L | 1.9 (2.3) | 2.0 (1.8) | 3.7 (3.0) | 2.4 (4.4) | 3.6 | 4.7 |
| n (%) |  |  |  |  |  |  |
| Corticosteroids^1^ | 5 (35.7) | 1 (2.3) | 6 (42.9) | 1 (4.2) | 1 | 0 |
| Hip fracture, relative^2^ | 1 (7.1) | 4 (9.1) | 3 (21.4) | 7 (29.2) | 0 | 1 |
| Smoking^3^ | 2 (14.3) | 12 (27.3) | 1 (7.1) | 4 (16.6) | 1 | 0 |
| Low physical activity ^4^ | 4 (28.6) | 27 (61.3) | 9 (64.3) | 14 (58.3) | 0 | 1 |

^1^ Regular for > 3 months, ^2^ First degree relative, ^3^ Including occasional, ^4^ < 2.5 h /week excluding walks

**Table S4.** Odds Ratios and Confidence Intervals for subgroups with low BMD in TH and/or LS

|  | | **T-score <-1.00 ─ >-2.5** | | | | **T-score ≤-2.5** | | | |
| --- | --- | --- | --- | --- | --- | --- | --- | --- | --- |
|  |  | **OR (95% CI) Unadj** | ***p*** | **OR (95% CI)**  **Adj** | ***P*** | **OR (95% CI) Unadj** | ***P*** | **OR (95% CI)**  **Adj** | ***P*** |
| Corticosteroids^1^ | SSc | 1.08 (0.54–2.16) | 0.8 | 1.26 (0.59–2.70) | 0.6 | 1.54 (0.61–3.90) | 0.4 | 2.26 (0.77–6.69) | 0.14 |
|  | Ctrl | 0.44 (0.12–1.69) | 0.2 | 0.33 (0.08–1.40) | 0.13 | 1.96 (0.50–7.66) | 0.3 | 1.23 (0.23–6.51) | 0.8 |
| Hip fracture, | SSc | **3.63 (1.18–11.2)** | **0.025** | 2.65 (0.83–8.44) | 0.1 | 2.52 (0.59–10.8) | 0.2 | 1.72 (0.36–8.34) | 0.5 |
| relative^2^ | Ctrl | 1.54 (0.94–2.52) | 0.086 | 1.26 (0.74–2.13) | 0.4 | 1.01 (0.42–2.44) | >0.9 | 0.85 (0.33–2.19) | 0.7 |
| Smoker^3^ | SSc | 1.75 (0.82–3.72) | 0.15 | 1.79 (0.69–4.07) | 0.2 | **3.61 (1.40–9.29)** | **0.008** | **3.42 (1.17–10.0)** | **0.025** |
|  | Ctrl | **0.56 (0.35–0.88)** | **0.013** | 0.71 (0.43–1.18) | 0.2 | 1.10 (0.56–2.18) | 0.8 | 1.46 (0.67–3.22) | 0.3 |
| Low physical | SSc | **1.89 (1.03–3.46)** | **0.039** | **2.09 (1.08–4.06)** | **0.029** | 1.52 (0.65–3.54) | 0.3 | 1.61 (0.62–4.20) | 0.3 |
| activity^4^ | Ctrl | 0.81 (0.56–1.18) | 0.3 | 0.86 (0.56–1.31) | 0.5 | 1.39 (0.75–2.58) | 0.3 | 1.61 (0.80–3.22) | 0.2 |
| CRP mg/L^5^ | SSc | 1.35 (0.99–1.84) | 0.058 | 1.40 (0.97–2.02) | 0.075 | **1.86 (1.22–2.82)** | **0.004** | **1.87 (1.15–3.03)** | **0.012** |
|  | Ctrl | 0.92 (0.74–1.13) | 0.4 | 0.94 (0.73–1.22) | 0.6 | **0.64 (0.43–0.96)** | **0.032** | 0.69 (0.42–1.11) | 0.13 |

Logistic regression analysis. Odds Ratios (ORs) and Confidence Intervals (CIs) for subgroups with low BMD in TH and/or LS with T-score <-1.00 ─ >-2.5 and T-score ≤-2.5 in patients with SSc and matched controls. Adjusted for sex, age, BMI and menopause. ^1^ Regular for > 3 months, **^2^** 1st degree relative, ^3^ Including occasional, ^4^ < 2.5 h /week (excluding walks), ^5^ Log of CRP mg/L

**Table S5.** Associations between T-score and putative risk factors for low BMD in SSc patients.

|  | Unadjusted hip | | | Unadjusted spine | | |
| --- | --- | --- | --- | --- | --- | --- |
|  | **B** | **95% CI** | ***p*** | **B** | **95% CI** | ***p*** |
| Disease duration (years) | -0.01 | -0.03, 0.01 | 0.4 | 0.00 | -0.02, 0.02 | >0.9 |
| Diffuse cSSc | -0.15 | -0.53, 0.23 | 0.4 | 0.22 | -0.26, 0.70 | 0.4 |
| Finger ulcers | -0.36 | -0.90, 0.18 | 0.2 | 0.02 | -0.66, 0.70 | >0.9 |
| Telangiectasia | -0.35 | -0.65, -0.04 | 0.026 | -0.18 | -0.57, 0.20 | 0.4 |
| Pitting scars | 0.04 | -0.27, 0.36 | 0.8 | -0.04 | -0.43, 0.35 | 0.8 |
| mRSS | 0.00 | -0.03, 0.02 | 0.8 | 0.00 | -0.03, 0.03 | 0.9 |
| Latest vital lung capacity (%) | 0.01 | 0.00, 0.02 | 0.086 | -0.01 | -0.02, 0.00 | 0.2 |
| Calcinosis on first hand x-ray | -0.27 | -0.61, 0.06 | 0.11 | -0.02 | -0.44, 0.40 | >0.9 |
| Antibodies |  |  |  |  |  |  |
| ACA | — | — |  | — | — |  |
| ARA | -0.20 | -0.75, 0.36 | 0.5 | -0.04 | -0.72, 0.65 | >0.9 |
| ATA | -0.23 | -0.72, 0.26 | 0.4 | 0.41 | -0.20, 1.0 | 0.2 |
| ANA pos (not ACA, ARA, ATA) | 0.17 | -0.19, 0.53 | 0.3 | 0.61 | 0.16, 1.1 | 0.008 |
| ANA neg | 0.35 | -0.25, 0.95 | 0.2 | 0.42 | -0.30, 1.1 | 0.3 |
| Corticosteroid duration (months) | 0.00 | 0.00, 0.00 | 0.14 | 0.00 | 0.00, 0.01 | 0.10 |
| P-CRP (log) (mg/L) | -0.30 | -0.44, -0.16 | <0.001 | -0.20 | -0.38, -0.02 | 0.029 |
| S-25-OH Vitamin D (nmol/L) | 0.00 | -0.01, 0.01 | 0.8 | 0.00 | -0.01, 0.01 | >0.9 |
| P-Albumin (g/L) | 0.06 | 0.01, 0.10 | 0.014 | 0.03 | -0.02, 0.09 | 0.2 |
| P-PTH (log) (pmol/L) | -0.64 | -0.99, -0.29 | <0.001 | -0.47 | -0.92, -0.02 | 0.042 |
| COMP (U/L) | 0.00 | -0.03, 0.03 | >0.9 | 0.02 | -0.02, 0.07 | 0.3 |
| P-NT-proBNP (log) (ng/L) | -0.42 | -0.55, -0.29 | <0.001 | -0.23 | -0.40, -0.06 | 0.009 |

Unadjusted linear regression analysis

**Supplementary Figure S1.** The predicted and observed total hip T-scores in SSc-patients. The regularized regression model, using elastic net in training and testing after calibration.

GAM Generalized Additive Model
